# Inhibition of the Renin-Angiotensin System Reduces Gene Expression of Inflammatory Mediators in Adipose Tissue Independent of Energy Balance

**DOI:** 10.3389/fendo.2021.682726

**Published:** 2021-06-02

**Authors:** Caitlin S. Mitchell, Shirmila D. Premaratna, Garth Bennett, Maria Lambrou, Lauren A. Stahl, Markandeya Jois, Elizabeth Barber, Christopher P. Antoniadis, Stephen C. Woods, David Cameron-Smith, Richard S. Weisinger, Denovan P. Begg

**Affiliations:** ^1^ School of Psychology, UNSW Sydney, Sydney, NSW, Australia; ^2^ Department of Animal, Plant and Soil Sciences, School of Life Sciences, La Trobe University, Melbourne, VIC, Australia; ^3^ School of Psychological Science, La Trobe University, Melbourne, VIC, Australia; ^4^ Department of Physiology, Anatomy and Microbiology, School of Life Sciences, La Trobe University, Melbourne, VIC, Australia; ^5^ Department of Nutrition, Dietetics and Food, Faculty of Medicine, Nursing and Health Sciences, Monash University, Melbourne, VIC, Australia; ^6^ Department of Psychiatry and Behavioral Neuroscience, University of Cincinnati, Cincinnati, OH, United States; ^7^ Singapore Institute for Clinical Sciences, Agency for Science, Technology and Research, Singapore, Singapore

**Keywords:** renin-angiotensin system antagonists, obesity, adipose tissue, inflammatory mediators, body weight, energy expenditure, gene expression

## Abstract

Obesity is a growing health problem worldwide. The renin-angiotensin system (RAS) is present in adipose tissue, and evidence suggests that it is involved in both diet-induced obesity and the inflammation associated with obesity. The present experiments determined the effect of (1) different angiotensin-converting enzyme (ACE) inhibitors (captopril, perindopril, enalapril) and angiotensin receptor blockers (ARBs: telmisartan, losartan) on adiposity of mice fed a high-fat diet for 28 days (2); acute treatment with the ACE-inhibitor captopril on gene expression of inflammatory markers in mice fed a high-fat diet (HFD); and (3) short-term (2 days) and chronic (28 days) treatment of ACE-inhibition on energy expenditure (EE) and energy balance in mice fed HFD *ad libitum* (AL), as well as receiving HFD limited to the amount of calories eaten by controls (pair-fed (PF) group). Body weight, food intake, adiposity and plasma leptin were lower in ACE inhibitor or ARB-treated groups over 28 days compared with HFD untreated mice. Short-term treatment with captopril led to increased EE relative to the level in the PF group. After 28 days, EE was lower in both captopril-treated and PF mice compared with AL, but the effect was greater in the captopril-treated group. Adiponectin was elevated in captopril-treated mice, but not in PF mice, after both 2 and 28 days. Additionally, acute RAS blockade in HFD-fed mice reduced mRNA expression for MCP-1, IL-6, TLR4, and leptin in adipose tissue relative to values in untreated groups. These data demonstrate that ACE inhibition and angiotensin receptor blockade reduce food intake to produce weight loss and suggest that the anti-inflammatory effects of ACE inhibition may be independent of weight loss.

## Introduction

The renin-angiotensin system (RAS) and its principal active component, angiotensin (ANG) II, have an important role in maintaining energy balance. Interest in the interactions of the RAS with energy balance arose from the identification of some components of the classical RAS within adipose tissue (1–3), and more recent *in vitro* data have identified a fully functional RAS within adipose tissue (4–7). Animals lacking components of the RAS have significant disruptions to energy homeostasis (8). For example, mice lacking genes for renin, angiotensinogen (AGT), angiotensin-converting enzyme (ACE), or angiotensin receptors (AT1) each have reduced body weight and resistance to high-fat diet (HFD)-induced weight gain (9–13). Analogously, compounds that inhibit RAS activity also reduce body weight. For example, ACE inhibition decreases body weight in rats (14–20), mice (21) and humans (22), while AT1 receptor antagonists reduce body weight, or limit weight gain, in rats (23–26), mice (27, 28) and humans (29).

Obesity is associated with increased inflammation in adipose tissue, with monocyte chemoattractant protein 1 (MCP-1), IL-6, toll-like receptor 4 (TLR4), and TNFα elevated in adipose tissue of obese relative to lean individuals (30). Obesity is also accompanied by changes in the circulating abundances of key adipokines involved in appetite regulation, including suppressed adiponectin and increased leptin concentrations (31). Adipose tissue is also important in the regulation of systemic RAS with increasing evidence that ANG II is an important contributing factor intersecting to heighten the inflammatory risks posed by obesity and cardio-metabolic disease (32–34). Although chronic treatment with an ACE inhibitor significantly reduces expression of inflammatory markers within adipose tissue (35), and has been shown clinically to alter adiponectin and leptin concentrations in hypertensive individuals (34), it is not clear whether these changes are mediated by ACE inhibition directly, or are secondary effect to the loss of body weight.

There are numerous ACE inhibitors and angiotensin receptor blockers (ARB), which have subtle differences in their mechanisms of action. For instance, enalapril (ENL) and perindopril (PER) are pro-drugs, which require liver metabolism for conversion into an active compound (36). ENL and PER are also longer acting that other ACE inhibitors, such as captopril (CAP) (37). Similarly, the ARB telmisartan (TEL) has a longer half-life and a higher affinity for the AT1 receptor compared to losartan (LOR) (38). Moreover, some ACE inhibitors cross the blood-brain barrier and act centrally, where they can inhibit ACE in the brain to affect central regulation of blood pressure (39). CAP and PER have modest, short-acting effects within the brain while ENL cannot access the central nervous system (39, 40). Interestingly, the pharmacokinetic and pharmacodynamic differences between these drugs do not alter their clinical efficacy, as all drugs act to either inhibit ACE or block the actions of ANG II at the receptor (36, 41).

The aim of the current experiments was to investigate the effects of RAS blockade on treatment of diet-induced obesity (DIO), energy expenditure (EE) and indicators of inflammation. Experiment 1 determined the specificity of the DIO-inhibition *via* RAS antagonism by comparing different ACE inhibitors and ARB. Having established that different forms of RAS blockade produce similar effects on body weight, body composition, and circulating levels of adiposity signals, Experiment 2 then examined the effects of acute administration of the ACE inhibitor, CAP, on mRNA expression of inflammatory markers in adipose tissue to determine if the previously observed decrease in inflammatory gene expression caused by chronic ACE inhibition (35) occurs prior to weight loss. Whilst, experiment 3 was conducted to assess the effects of short (2 days) *vs.* longer (28 days)-term ACE inhibition by CAP on EE, with the hypothesis that CAP would increase both adiponectin and metabolic rate at both durations.

## Materials and Methods

### Animals

Male C57BL/6J mice (n = 180; Monash Animal Services, Clayton, VIC, Australia and Animal Resource Centre, Canning Vale, WA, Australia) were housed in the La Trobe University central animal facility and given at least one week to acclimate prior to experimentation. Animals were single-housed and maintained on a 12:12-h reverse light/dark cycle with temperature maintained at 22 ± 3°C. Mice 6 to 8 weeks of age were provided a pelleted HFD (21% fat w/w; Speciality Feeds, Australia) with *ad libitum* water except where specified. There was a small variation among studies on some measures due to availability of mice at the time of purchase, but none that altered any conclusion. All animal procedures were approved by the Animal Ethics Committee of La Trobe University (approval nos. AEC09-16-P and AEC10-04-P) or the UNSW Animal Care and Ethics Committee (approval no. 15/8B).

### Design

#### Experiment 1

Mice 8 weeks of age were randomly assigned to one of six conditions (n=12/group) assessing different ACE inhibitors (CAP, ENL, PER) and ARB (TEL, LOS). The HFD was available *ad libitum* and contained CAP (100 mg/kg with an average dose of 13.3 mg/kg/d), ENL (33 mg/kg with an average dose of 4.4 mg/kg/d), TEL (66 mg/kg with an average dose of 8.8 mg/kg/d), PER (8 mg/kg with an average dose of 1.1 mg/kg/d), LOS (83 mg/kg with an average dose of 11.1 mg/kg/d) (Sigma-Aldrich, St. Louis, MO) or nothing-added [control (CON)]. Doses were chosen based on recommended prescribed intakes for human hypertension (42), relative to our established dose of CAP (21). Various ACE inhibitors and ARB were used in this study to confirm that observed metabolic and behavioral effects are not specific to any one drug. Food intake, water intake and body weight were measured at baseline and every 7 days for 28 days. At the conclusion of the experiment, mice were fasted for 2 h and anesthetized (Ketamine 60 mg/kg and Xylazine 8 mg/kg; ip; 0.2 mL), and body composition was determined using DEXA (pDEXA Sabre, Norland Medical Systems). Blood was collected by cardiac puncture with a 25-gauge needle attached to an EDTA-treated syringe. Blood samples were centrifuged for 12 min at 14,100 rcf and stored at −20°C.

#### Experiment 2

Mice 8 weeks of age were fed HFD for 6 weeks *ad libitum*. They were then fasted overnight (12 h) and randomly assigned to Fasted, CON or CAP groups (n=6/group). The Fasted group did not have food or fluid returned, the CON group had food and water returned for 3 h and the CAP group had food and water containing CAP (0.05 mg/mL) returned for 3 h (mean intake 2.11 ± 0.24 mg/kg). After 3 h mice in all groups were sacrificed by the method described for Experiment 1 with the omission of the DEXA scan. Body weight and food and fluid intake were measured. Epididymal fat was harvested, snap-frozen in liquid nitrogen and stored at −80°C.

#### Experiment 3

On Day 1, mice 6 weeks of age were placed in a Labmaster indirect calorimetry system for acclimation. On Days 2 and 3 in the calorimeter they were randomly assigned to a control group with *ad libitum* food (AL; n = 12), a CAP-treated group (0.05 mg/mL drinking water) with *ad libitum* food (CAP; n=12), or a group pair-fed to the CAP group (PF; n=12). The final day (Day 3) was used for analyses. An additional cohort of mice was moved to the calorimetry apparatus after 25 days of AL (n=12), CAP (n=12) or PF (n = 12) treatment; i.e., there were 25 days of body weight and ingestive behavior measures on HFD prior to the calorimetric analyses in this cohort. Upon removal from calorimetry apparatus, mice were sacrificed in the manner described in Experiment 1.

### Plasma Hormone Measurements

Plasma adiponectin and leptin concentrations were measured using ELISA kits (EZML-82K Mouse Leptin ELISA and EZML-60K Mouse Adiponectin ELISA, Linco Research, St Charles, MO). Absorbance was read at 450 nm and 590 nm for all assays using a microplate spectrophotometer (Molecular Devices Spectra Max 250, GMI, Ramsey, MN).

### mRNA Expression

Total RNA was extracted from ~100 mg of adipose tissue using Tri-reagent (PE Applied Biosystems, CA, USA). MCP-1, IL-6, TLR4, leptin, and adiponectin (GeneWorks Pty Ltd, Hindmarsh, SA, Australia; see **Supplementary Table S1**) mRNA expression were quantified after reverse transcription using quantitative real-time PCR on a fluorometric thermal cycler (7500 Fast Real-Time PCR System, PE Applied Biosystems) and Power SYBR^®^ Green PCR Master Mix (PE Applied Biosystems). All results were normalized to levels of 28S ribosomal RNA. The oligonucleotide sequences have been provided previously (35).

### Indirect Calorimetry and General Locomotor Activity (GLA)

Mice were placed in the calorimetry cages for 3 days; the first 2 days were considered the acclimation phase, and data were analyzed only for the final 24 h. The system used was a custom-built, four-cage, open-circuit calorimeter (LabMaster; TSE Systems GmbH, Bad Homburg, Germany). GLA was also continuously monitored as movement counts. Dependent variables were total EE during the light and dark phases, respiratory quotient (RQ) and GLA.

### Statistical Analysis

Data were analyzed by a one, two-way analysis of variance (ANOVA) or two-way repeated measures ANOVA, followed by *post hoc* least significant difference tests following significant interaction effects (Statistica 7, StatSoft, Tulsa, OK). Statistical significance was accepted at *P* < 0.05, two-tailed. Results are reported as mean ± SEM.

## Results

### Experiment 1

#### Food and Water Intake

**Figure 1:**
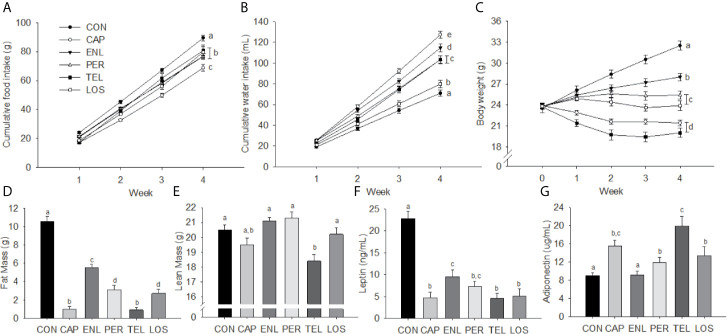
Inhibition of the RAS decreases food intake, body weight, body fat and plasma leptin. Food intake **(A)**, water intake **(B)**, body weight **(C)**, fat mass **(D)**, lean mass **(E)**, leptin **(F)** and adiponectin **(G)** in mice treated with angiotensin-converting enzyme (ACE) inhibitors (Captopril [CAP], enalapril [ENL], perindopril [PER]) or angiotensin receptor antagonists (ARBs: telmisartan [TEL], losartan [LOS]). Data are represented as mean ± SEM with significant differences indicated at the p<0.05 by differing superscript letters.

Over 28 days of HFD feeding, groups treated with an ACE inhibitor or an ARB had lower food intake than untreated CON [F(15, 195)=4.331, p<0.001], see **Figure 1A**. Cumulative food intake was lower than CON in CAP (p<0.01), LOS (p<0.05) and TEL (p<0.01) during Week 1, ENL (p<0.05) by Week 2 and PER by Week 3 of treatment (p<0.01), and all group intakes remained significantly lower than CON at Week 4. Food intake in the CAP group was significantly lower than that of all other groups by Week 3 (ps<0.05). Total water intake differed among groups [F(5, 49)=31.635, p<0.001]; intakes were lowest in CON (p<0.05 *vs.* all groups) and greatest in CAP (p<0.01 *vs.* all groups). LOS animals had lower water intake than all groups other than CON (ps<0.01), see **Figure 1B**. It is important to note that, when considering data from earlier publications, we have now made a dozen or more independent replications of the same basic experiment and had comparable results in all of them (20, 21, 35, 43).

#### Body Weight and Body Composition

Starting weights did not differ among the groups whereas after 4 weeks of treatment there was a significant effect of group [F(20, 260)=39.134, p<0.001]. Weight gain in CON over 28 days was 9.01 ± 0.76 g. Weight gains in ENL (4.17 ± 0.43 g), PER (1.61 ± 0.57 g) and LOS (0.15 ± 0.92 g) animals were each lower relative to CON. Both CAP (−2.48 ± 0.50 g) and TEL (−3.77 ± 0.77 g) lost weight over the course of the experiment. Weekly body weights are depicted in **Figure 1C**.

There were significant main effects for both fat mass [F(5, 65)=79.442, p<0.001] and lean mass [F(5, 65)=9.602, p<0.001]. Fat mass was greater in CON animals compared with all other groups (ps<0.001). ENL mice had 53% of the fat mass of CON animals and the CON group maintained a significantly greater fat mass than all other treatment groups (ps<0.001). CAP and TEL animals had lower fat mass than the remaining groups (ps<0.01), see **Figure 1D**. Lean-mass was lower only in TEL animals compared with all other groups other than CAP (ps<0.01), see **Figure 1E**. Fat and lean mass were calculated as percentage of body weight for all treatment groups (**Table 1**).

**Table 1:** Fat mass and lean mass calculated as a percentage of body weight.

	CON	CAP	ENL	PER	TEL	LOS
Fat mass (%)	10.6	1	5.5	3.1	0.9	2.7
Lean mass (%)	20.5	19.5	21.1	21.3	18.4	20.2

Values calculated from mean of each treatment group.

CON, control; CAP, captopril; ENL, enalapril; PER, perindopril; TEL, telmisartan; LOS, losartan.

#### Plasma Hormones

Plasma leptin was elevated in CON animals [F(5, 28)=24.475, p<0.001] relative to all other groups (ps<0.001) and ENL-treated mice had higher leptin relative to CAP, TEL and LOS mice (ps<0.05), see **Figure 1F**. There was a significant treatment effect on adiponectin [F(5, 28)=8.017, p<0.001], see **Figure 1G**, with TEL having higher adiponectin relative to all groups other than CAP (ps<0.05), and CAP, PER and LOS treatment having increased adiponectin relative to CON (ps<0.05).

### Experiment 2

#### Ingestive Behaviors and Body Weight

There were no differences in body weight, food or fluid intakes among treatment groups at 3 h post-treatment, see **Table 2**.

**Table 2:** Food intake, fluid intake, final body weight, liver and epididymal fat weights.

	Overnight Fast	Re-Fed CON	Re-Fed CAP
**Food intake (g)**	–	1.13 ± 0.10	0.87 ± 0.10
**Fluid intake (mL)**	–	1.55 ± 0.09	1.34 ± 0.16
**Final body weight (g)**	30.92 ± 2.15	31.38 ± 1.11	31.53 ± 1.30
**Liver weight (g)**	1.37 ± 0.14	1.50 ± 0.16	1.56 ± 0.13
**Epididymal fat weight (g)**	1.38 ± 0.21	1.41 ± 0.17	1.34 ± 0.11

Values are mean ± SEM.

#### Adipose Tissue mRNA

**Figure 2:**
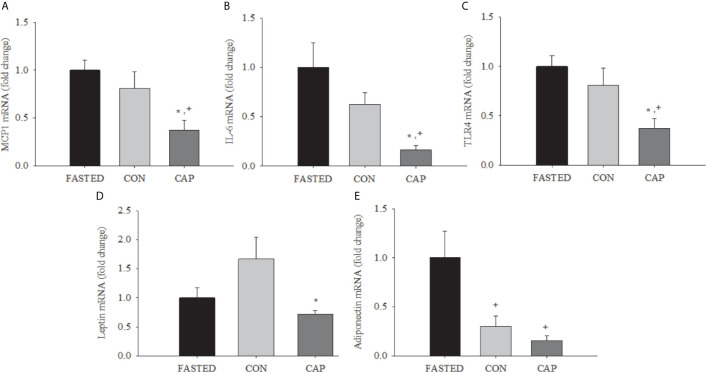
Acute captopril treatment decreases adipose tissue mRNA expression of inflammatory mediators and adipokines. MCP-1 **(A)**, IL-6 **(B)**, **(C)** TLR4, leptin **(D)**, and adiponectin **(E)** in epididymal adipose tissue (* p<0.05 *vs.* Fasting, ^+^ p< 0.05 *vs.* CON). Results are normalized to the expression of 28s ribosomal RNA.

As depicted in **Figure 2**, at 3 h post-treatment the expressions of mRNA for MCP-1, IL-6, and TLR4 were lower in the adipose tissue of CAP-treated mice compared with those of CON and FASTED animals (p<0.05); CAP-treated mice also had lower leptin mRNA expression compared to CON (p<0.05). CAP and CON mice had lower adiponectin mRNA expression relative to FASTED animals (p<0.05). The mean cycle threshold (CT) values were not significantly different between treatment groups (see **Supplementary Table S2**).

### Experiment 3

#### Food and Fluid Intake (2-Day Treatment)

**Figure 3:**
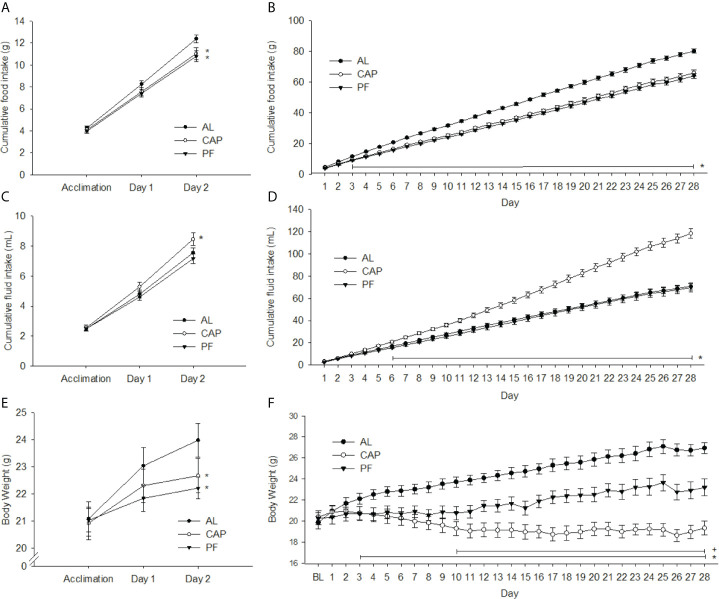
2 or 28 days of captopril treatment decreases food intake and body weight of mice relative to *ad libitum*-fed and pair-fed controls. Food intake **(A, B)**, water intake **(C, D)** and body weights **(E, F)** of ad libitum-fed (AL) controls, captopril (CAP) treated, and pair-fed (PF) animals, treated for 2 or 28 d, respectively. Data are represented as mean ± SEM with significant differences indicated at the p<0.05 level * *vs.* AL, ^+^ *vs.* PF. The two adjacent asterisks are referring to distinct significant differences shown in two different treatment groups.

There was a significant interaction effect (treatment group x day) on cumulative food intake [F(4, 64) = 3.02, p<0.05]. As depicted in **Figure 3A**, food intake was lower in CAP (p<0.01) and PF (p<0.01) compared with levels in AL animals on Day 2 of treatment. There was also a significant interaction for cumulative water intake [F(4, 62) = 3.25, p<0.05]; water intake was increased in CAP relative to AL (p<0.05) and PF (p<0.01) groups by Day 2 of treatment, see **Figure 3C**. There was no difference in fluid intake between the AL and PF groups.

#### Food and Fluid Intake (28-Day Treatment)

Over the 28-day treatment period cumulative food intake differed significantly among treatment groups [F(54, 891)=16.09, p<0.001]. Differences were observed between AL and the CAP/PF groups from Day 3 (p<0.05) through Day 28 (p<0.001), see **Figure 3B**. Cumulative water intake was greater in CAP animals than in AL/PF groups from Day 6 [F(54, 891)=53.47, p<0.001], and no differences were observed between AL and PF groups, see **Figure 3D**.

#### Body Weight, Body Composition and Tissue Weights (2-Day Treatment)

There was a significant interaction effect (treatment group x day) on body weight [F(4, 66)=5.67, p<0.001], see **Figure 3E**. Body weights of both CAP (p<0.05) and PF (p<0.01) mice were significantly lower compared with those of AL mice on Day 2 of treatment, with no significant difference between CAP and PF animals. The DEXA analyses revealed no differences in total fat or lean mass, see **Table 2**. Dissected liver weights were lower in CAP (p<0.05) and PF (p<0.05) compared with AL mice [F(2, 33)=3.29, p<0.05], whereas there was no difference in epididymal adipose tissue weight.

#### Body Weight, Body Composition and Tissue Weights (28-Day Treatment)

There was a significant interaction effect over the treatment period (treatment group x day) on body weight [F(56, 924)=23.91, p<0.001]. Body weights of both CAP (p<0.05) and PF (p<0.05) groups were lower compared with weights of AL from Day 3 of treatment and remained lower for the duration of the experiment. CAP-treated mice weighed significantly less than PF mice after 10 days of treatment (p<0.05) and this continued through to completion of the experiment at 28 days, see **Figure 3F**. Fat mass was lower in CAP (p<0.001) and PF (p<0.01) mice compared with that in AL [F(2, 33)=31.25, p<.001], CAP was also lower than PF (p<0.01). Total lean mass was lower in CAP relative to both PF (p<0.05) and AL (p<0.001) mice [F(2, 33)=8.24, p<0.001], see **Table 3**.

**Table 3:** Body composition, liver and epididymal fat weights after 2 days or 28 days of captopril (CAP) treatment.

	AL	CAP	PF
**2 days**			
Body Fat (g)	3.61 ± 0.30	3.57 ± 0.39	3.60 ± 0.32
Lean mass (g)	18.84 ± 0.30	17.60 ± 0.31	17.28 ± 0.26
Liver weight (g)	1.33 ± 0.06	1.15 ± 0.06*	1.12 ± 0.06*
Epididymal fat (g)	0.41 ± 0.03	0.39 ± 0.03	0.38 ± 0.02
**28 days**			
Body Fat (g)	6.58 ± 0.48	1.19 ± 0.31*	3.65 ± 0.61*^,+^
Lean mass (g)	18.49 ± 0.30	16.60 ± 0.42*	17.75 ± 0.24*^,+^
Liver weight (g)	1.30 ± 0.05	0.77 ± 0.03*	0.93 ± 0.06*^,+^
Epididymal fat (g)	0.85 ± 0.06	0.27± 0.03*	0.50 ± 0.06*^,+^

Values are mean ± SEM.

*Significant difference from ad libitum (AL).

^+^Significant difference between CAP and pair-fed (PF).

Liver weights were significantly lower in CAP mice compared with PF (p<0.05) and AL (p<0.001) mice, and liver mass was also lower in PF compared with AL mice (p<0.01) [F(2, 33)=13.1, p<0.001]. Epididymal fat mass differed among all groups [F(2, 33)=32.364, p<0.001]; with CAP less than PF (p<0.01) and AL (p<0.001), and PF less than AL (p<0.001), see **Table 3**.

#### Energy Expenditure (EE), Respiratory Quotient (RQ) and General Locomotor Activity (GLA) (2 Days)

**Figure 4:**
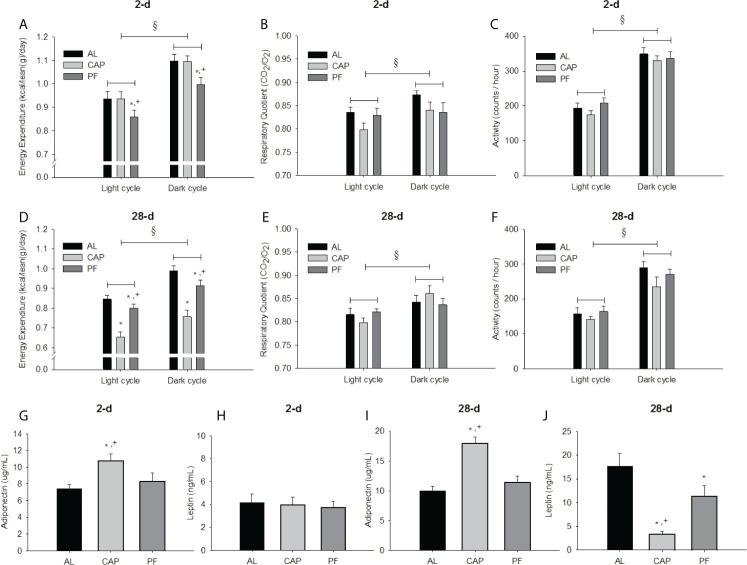
Energy expenditure, respiratory quotient, locomotor activity, and plasma adipokines of mice treated with captopril for 2 or 28 days with both *ad libitum-* and pair-fed controls. Energy expenditure **(A, D)**, respiratory quotient **(B, E)**, activity **(C, F)**, plasma adiponectin **(G, I)** and leptin **(H, J)** of ad libitum-fed (AL) controls, captopril (CAP)-treated, and pair-fed (PF) animals, treated for 2 or 28 days, respectively. Data are represented as mean ± SEM with significant differences indicated at the p<0.05 level * *vs.* AL, ^+^ between CAP and PF, § between light phase.

There was no significant interaction of treatment group x light cycle on EE. There was, however, a significant main effect of treatment group overall [F(2, 30)=3.52, p<0.05]. Post hoc analyses revealed that PF mice had significantly lower EE than both CAP and AL groups (p<0.05), see **Figure 4A**. There was a significant increase in EE during the dark cycle regardless of group [F(1, 30)=408.53, p<0.001]. RQ and GLA did not provide significant interactions of treatment group x light cycle. The only differences observed were increases in RQ [F(1, 30)=16.37, p<0.001] and GLA [F(1, 30)=150.89, p<0.001] during the dark cycle, and this occurred regardless of group, see **Figures 4B, C**.

#### Energy Expenditure (EE), Respiratory Quotient (RQ) and General Locomotor Activity (GLA) (28 Days)

There was a significant main effect of treatment group on EE [F(2, 33)=21.63, p<0.001]. Both CAP (p<0.001) and PF (p<0.05) animals had significantly lower EE than AL, and CAP animals had lower EE than PF animals after 28 days of treatment (p<0.001), see **Figure 4D**. There was a significant increase in EE in the dark cycle regardless of group [F(1, 33)=89.991, p<0.001]. No significant interaction effect of treatment group x light cycle on EE was observed.

There was a significant interaction effect of treatment group x light cycle on RQ [F(2, 33)=4.31, p<0.05]. Increases in RQ were observed from light to dark in CAP (p<0.001) and AL (p<0.05) mice, but not in PF, see **Figure 4E**. The only differences observed in GLA were during the dark cycle and occurred regardless of treatment group, see **Figure 4F**.

#### Plasma Hormones (2-Day Treatment)

Plasma adiponectin was significantly different among treatment groups [F(2, 19)=4.21, p<0.05]. CAP had higher adiponectin than both PF (p<0.05) and AL (p<0.05). Plasma leptin did not differ among groups, see **Figures 4G, H**.

#### Plasma Hormones (28-Day Treatment)

Plasma adiponectin differed among treatment groups [F(2, 33)=18.75, p<0.001]. CAP had higher adiponectin than both PF (p<0.001) and AL (p<0.001), with no difference between PF and AL mice, see **Figure 4I**. Plasma leptin was also significantly different among groups [F(2, 21)=12.66, p<0.001]. CAP (p<0.001) and PF (p<0.05) mice had lower leptin levels compared with AL, and CAP animals also had lower plasma leptin than PF animals (p<0.01), see **Figure 4J**.

## Discussion

In Experiment 1, mice given *ad libitum* access to a HFD supplemented with an ACE-inhibitor or with an ARB had substantially lower body weight, food intake and adiposity compared with HFD-fed, untreated mice. This suggests that the observed effects are primarily related to reducing peripheral angiotensin signaling, rather than to the previously hypothesized increase in central RAS activity (8, 44). Although the outcomes of previous reports of ACE inhibitors and ARBs on weight loss are not consistent, most rodent research has found either weight loss or inhibition of weight gain (see (45) for a review). The ACEs and ARBs in Experiment 1 were added to the food pellets at levels that assumed that a 30 g mouse eats approximately 4 g of food/d. The actual doses chosen were then calculated based on recommended human doses adjusted for differences in the metabolic rate of mice. While the prevention of the RAS activity in all groups would be expected to be similar, differential outcomes among groups may be due to differences in the pharmacokinetics and pharmacodynamics of the compounds in the mouse (46). However, and importantly, all of these compounds produced a reduction in weight gain and adiposity, such that the minor differences observed are most likely related to dose effects. Further, there were no instances of mortality or illness in any group, implying that they were well-tolerated. As expected, ACE inhibitors increased water intake. The mechanism for this has been discussed previously (47) and appears to be due to increased conversion of ANG I to ANG II within the brain. Interestingly, the ARBs LOS and TEL also caused increased water intake. The increase was evident by Week 3 of treatment and may be a function of the increase in plasma renin associated with AT_1_ blockade (48).

We used only mice fed a HFD, but previous studies investigated the effects of RAS inhibition in low fat-fed (or normal diet) animals; ACE-inhibition lowered body mass gain, food intake and fat mass compared with untreated controls (15, 17, 23). Therefore, given that the present experiments aimed to investigate the role of RAS blockade in the prevention of HFD-induced impairments, we used untreated HFD-fed mice as the appropriate control.

In HFD-fed mice, acute treatment with CAP in the drinking water for 3 h significantly lowered mRNA expression of the inflammatory mediators, MCP-1, IL-6, TLR4, and leptin compared with controls, despite not effecting food intake over this time. We have previously found that gene expression related to inflammation, including MCP-1, IL-6, and TLR4, is reduced by chronic administration of an ACE inhibitor (35). In DIO rodents, treatment with an ARB reduces white adipose tissue mRNA expression of TNF-alpha (23, 49), MCP-1 and IFN-gamma (50). Anti-inflammatory effects have also been observed following RAS blockade in agouti and leptin receptor deficient obese mouse models (51, 52). However, because the low-grade inflammatory state, that is mediated by adipokine secretion from adipocytes and adipose-resident macrophages, decreases with reduction in adipose depot mass, the data previously presented were confounded by the weight loss that had occurred in the ACE inhibitor-treated animals relative to the obesity observed in the control animals. Treatment of adipocytes in culture with an ARB decreased mRNA expression of adipokines related to inflammation, including RBP4, resistin, and visfatin (53), as well as pro-inflammatory cytokines IL-6 and TNF-alpha (50, 54, 55). The data in Experiment 2 indicate that acute RAS blockade *in vivo* significantly reduces gene expression of markers of inflammation prior to reductions in food intake or body weight.

The mechanism by which ACE inhibition suppresses inflammation remains unclear. As obesity develops, adipocytes begin to secrete low levels of TNFα, and TNFα in turn stimulates pre-adipocytes to produce MCP-1 (56). MCP-1 is a chemoattractant which facilitates recruitment of macrophages into adipose tissue (55, 57), and IL-6 production by macrophages is increased in adipose tissue in obesity (58). In addition, it has been suggested that IL-6 is involved in insulin resistance and its complications (58). TLR4 is a cell-surface receptor that produces innate immune responses to pathogens by inducing signaling cascades of kinase and transcription factor activation (59). These cascades lead to the production of pro-inflammatory cytokines and chemokines, including IL-6, eicosanoids and reactive oxygen species (ROS), all of which are effectors of immunity (60). Further, TLR4 is expressed in many insulin target tissues including liver, adipose tissue, skeletal muscle, vasculature, pancreatic β cells and brain (61). Thus, activation of TLR4 can inhibit insulin action directly *via* activation of pro-inflammatory kinases and ROS, and indirectly *via* activation of cytokine signaling cascades and systemic release of pro-inflammatory and insulin desensitizing factors (61). Overall, the present data suggest that ACE inhibition suppresses DIO-induced inflammation in adipose tissue independent of body weight, implying that the RAS itself may mediate parts of the inflammatory response to DIO.

Experiment 3 demonstrates that short-term (2 days) inhibition of ACE with captopril leads to a rapid reduction of food intake in mice fed a HFD. However, despite the reduction in energy intake, CAP-treated animals maintained EE at the level of AL animals, with EE of both CAP and AL groups being significantly greater than that of PF animals. The mechanism by which CAP reduced body weight gain despite the mice having similar food intake and lower EE is not clear and awaits future research; it is possible that a different dose of CAP might have led to a different outcome, but the dose we used (100 mg/kg of diet), and assuming a mouse consumes around 4 g of food/d which equates to 0.4 mg/day, translates to 13.3 mg/kg/day. This is in the center of the range suggested to be appropriate for mice (52).

An early increase in plasma adiponectin was also observed. Adiponectin has previously been reported to stimulate EE and thermogenesis after entering the CNS (62), and this may explain the ability of ACE inhibitor-treated animals to maintain EE despite a reduction in energy intake. Alternatively, given that ACE inhibition reduces peripheral ACE higher levels of circulating ANG I which is converted to ANG II at the circumventricular organs occurs, consequently elevating central levels of ANG II (47). Increased water intake following ACE inhibition is mediated by this elevated central activity of ANG II. Further, increased central ANG II signaling influences sympathetic activity, such that the temporary increase of EE observed in ACE inhibitor-treated mice may be partly mediated through increased sympathetic outflow to brown adipose tissue (44).

In mice consuming HFD, longer-term treatment with an ACE inhibitor (28 days) markedly lowered body weight compared to both AL and PF animals, and this difference was reflected in lower adiposity, liver weight and epididymal fat weight. After 28 days, EE in PF animals was lower than that of AL mice, and was even lower in the ACE inhibitor-treated group. Similarly to what occurred after the acute (2 days) ACE inhibition, adiponectin was significantly increased in ACE inhibitor-treated animals after 28 days compared with levels in the AL and PF groups. Leptin levels were consistent with the body weight and fat data, with AL animals having the highest levels followed by PF and then ACE inhibitor-treated mice. Thus, it would appear that once energy stores have been depleted, ACE inhibitor-treatment no longer has the ability to maintain EE at the level seen in AL or even PF mice. Interestingly, by 28 days, and despite the food intakes being equal for the PF and ACE inhibitor-treated animals, body weight of the ACE inhibitor-treated animals was 15% lower than that of the PF animals. The lower body weight was reflected in the lower EE, but the latter was insufficient to restore body weight to the level of the PF animal.

As reported previously (21), plasma adiponectin was increased and plasma leptin was lower following chronic treatment with an ACE inhibitor in mice fed a HFD than in untreated controls. The current study narrowed the time course of the changes in these hormones; i.e., adiponectin was elevated after just 2 days of ACE inhibition, and the increase persisted over 28 days. Since mRNA expression of adiponectin was not changed after 3 h of ACE inhibitor-treatment, greater than 3 h of ACE inhibition (but less than 2 days) appears necessary to change adiponectin regulation. Adiponectin is reduced in obesity (63) and increased with caloric restriction (64). The increased body weight of PF animals over the course of the 28 days indicates that the PF animals were receiving sufficient calories to maintain a positive energy balance, which would likely have limited any increase of adiponectin.

Leptin primarily acts as an adiposity signal, circulating in direct proportion to fat stores. Leptin is also associated with the low-grade inflammatory state in obesity (65). An increased inflammatory response occurs with the hyperleptinemia (66), and leptin is capable of controlling TNFα production and activation of macrophages (67). However, our data indicate that inflammatory markers in ACE inhibitor-treated animals are lower prior to a reduction in leptin expression, suggesting that leptin is not the principal factor in inflammation in obesity and its reduction by ACE inhibition.

Collectively, these studies improve our understanding of the time-course of prevention of diet-induced obesity by antagonism of the RAS. Indeed, within the first days of treatment with ACE inhibitor-treatment there is a decrease in food intake; however, EE is maintained, and actually elevated when compared with levels in animals consuming the same reduced amount of food but without ACE inhibition. However, after 28 days of treatment EE was significantly decreased in ACE inhibitor-treated animals relative to both AL and PF animals. This, along with increased adiponectin, produces a similar phenotype as a starvation response and is not observed in PF animals, indicating that the weight loss mediated by ACE inhibition is not a function of lowered energy intake alone.

## Data Availability Statement

The raw data supporting the conclusions of this article will be made available by the authors, without undue reservation.

## Ethics Statement

The animal study was reviewed and approved by Animal Ethics Committee of La Trobe University (Approval Nos. AEC09-16-P and AEC10-04-P) or the UNSW Animal Care and Ethics Committee (Approval No. 15/8B).

## Author Contributions

DB, RW, DC-S, and MJ designed the experiments. SP, EB, ML, GB, LS, and DB collected and analyzed the data. CM, DC-S, SW, and DB wrote the manuscript. All authors reviewed and edited the manuscript. All authors contributed to the article and approved the submitted version.

## Funding

This work was supported by the Australian Research Council (DE160100088 and DP170100063), the National Health and Medical Research Council APP1156622, and a Ramaciotti Foundation Establishment Grant.

## Conflict of Interest

The authors declare that the research was conducted in the absence of any commercial or financial relationships that could be construed as a potential conflict of interest.
