# Supplementary material for: Inhibition of the Renin-Angiotensin System Reduces Gene Expression of Inflammatory Mediators in Adipose Tissue Independent of Energy Balance
Source: Front Endocrinol (Lausanne). 2021 Jun 2;12:682726. doi: 10.3389/fendo.2021.682726 (PMC8206808; doi:10.3389/fendo.2021.682726)
Supplement: Supplementary file 1 [file Table_1.docx]

**S1 Table. Gene specific primer sequences used for quantitative reverse transcription PCR (RT-qPCR).**

| **Gene** | **Forward Primer (5’-3’)** | **Reverse Primer (3’-5’)** | **NCBI Reference Sequence** |
| --- | --- | --- | --- |
| 28s rRNA | CCATATCCGCAGCAGGTCTCC | CCCAGCCCTTAGAGCCAATCC | NR_003279.1 |
| MCP-1 | CTTCCTCCACCACCATGC | CCAGCCGGCAACTGTGA | NM_011333.3 |
| IL-6 | ACAAGTCGGAGGCTTAATTAC | TTGCCATTGCACAACTCTTTC | NM_031168.1 |
| TLR-4 | CCTGACACCAGGAAGCTTGAA | TCTGATCCATGCATTGGTAGGT | NM_021297.2 |
| Leptin | TCCAGAAAGTCCAGGATGACAC | CACATTTTGGGAAGGCAGG | NM_008493.3 |
| Adiponectin | AAGGACAAGGCCGTTCTCT | TATGGGTAGTTGCAGTCAGTTGG | NM_009605.4 |
