# Supplementary material for: Inhibition of the Renin-Angiotensin System Reduces Gene Expression of Inflammatory Mediators in Adipose Tissue Independent of Energy Balance
Source: Front Endocrinol (Lausanne). 2021 Jun 2;12:682726. doi: 10.3389/fendo.2021.682726 (PMC8206808; doi:10.3389/fendo.2021.682726)
Supplement: Supplementary file 2 [file Table_2.docx]

**S2 Table. Mean 28s CT for all animals in each group**

|  | **Overnight Fast** | | **Re-Fed CON** | **Re-Fed CAP** |
| --- | --- | --- | --- | --- |
|  | 12.55 | | 9.97 | 9.83 |
|  | 8.58 | | 10.39 | 9.61 |
|  | 10.74 | | 10.13 | 11.90 |
|  | 9.97  11.31  9.50 | | 10.65  9.48  12.74 | 10.14  9.81  10.16 |
| **Mean** |  | **10.44 10.56** | | **10.24** |
